# Epidemiological Characteristics and Mortality Predictors of Candidemia Due to *Candida albicans*: A Single-Center Experience from Türkiye

**DOI:** 10.3390/jof11110788

**Published:** 2025-11-02

**Authors:** Nevzat Ünal, Ayşe Sultan Karakoyun, İlker Ünal, Tuba Turunç, Cornelia Lass-Flörl, Macit Ilkit

**Affiliations:** 1 Division of Mycology, Department of Microbology, Faculty of Medicine, Çukurova University, Adana 01330, Türkiye; drnevzatunal@gmail.com; 2 Laboratory of Medical Microbiology, Adana City Training and Research Hospital, University of Health Sciences, Adana 01370, Türkiye; 3 Clinic Microbiology Laboratory, Dr Yusuf Azuzoğlu Silvan State Hospital, Silvan 21640, Türkiye; md.aysesultank@gmail.com; 4 Department of Biostatistics, Faculty of Medicine, Çukurova University, Adana 01330, Türkiye; ilkerun@cu.edu.tr; 5 Department of Infectious Diseases and Clinical Microbiology, Adana City Training and Research Hospital, Adana Faculty of Medicine, University of Health Sciences, Adana 01370, Türkiye; tubaturunc@yahoo.com; 6 Institute of Hygiene and Medical Microbiology, Medical University of Innsbruck, Excellence Center for Medical Mycology (ECMM), 6020 Innsbruck, Austria; cornelia.lass-floerl@i-med.ac.at

**Keywords:** *Candida albicans*, candidemia, epidemiology, fluconazole, mortality, risk factors

## Abstract

Candidemia is a major global health challenge, with *Candida albicans* being the most common causative agent. Since antifungal drugs may promote resistance, regular epidemiological studies are required. Nonetheless, microbiological and clinical data on *C. albicans* in Türkiye are limited. Therefore, we assessed data from *C. albicans* cases in a tertiary care hospital in Türkiye. Among 171 enrolled patients, the overall mortality rate was 66.7%. Univariate analysis showed that age, intensive care unit (ICU) admission, central venous catheterization, mechanical ventilation, hemodialysis, diabetes mellitus, Coronavirus disease 2019 (COVID-19) infection, steroid use, and hyperalimentation were associated with mortality. Multivariate logistic regression showed that age, ICU admission, steroid use and hyperalimentation were independently associated with mortality. In Cox regression, age, ICU admission, prior antifungal use, and absence of antifungal treatment after candidemia were independently associated with decreased survival. Fluconazole (FLC) was the most frequently used antifungal, and patients treated with FLC + amphotericin B or FLC + echinocandin had the best survival rates. All 171 isolates were susceptible to all tested antifungals. Our findings show high mortality rates and reveal mortality-associated factors. *Candida albicans* remains susceptible to all antifungals; therefore, timely diagnosis and appropriate antifungal treatment can enhance survival and clinical outcomes.

## 1. Introduction

Candidemia is one of the most important nosocomial bloodstream infections caused by *Candida* species, with approximately 1.5 million cases of invasive candidiasis or candidemia occurring globally each year, nearly 1 million of which result in death, with mortality rates exceeding 60% [1]. *Candida albicans* accounts for 40–60% of cases [2,3]. *Candida parapsilosis*, *C. tropicalis*, *C. glabrata*, and *C. krusei* are the most common non-*albicans* species, with prevalence varying by geographical region [4,5,6]. As species distribution and antifungal resistance patterns can vary regionally, local epidemiological data are critical for clinical management [4,7].

The Coronavirus disease 2019 (COVID-19) pandemic, a global health crisis, has significantly changed the epidemiology of candidemia. Patients with severe COVID-19 admitted to the intensive care units (ICUs) frequently require prolonged hospitalization, broad-spectrum antibiotic therapy, corticosteroid or immunomodulatory therapies, and invasive medical procedures such as placement of a central venous catheter (CVC), mechanical ventilation, and hemodialysis [8,9,10]. Collectively, these factors predispose patients to secondary fungal infections. Among these, candidemia, particularly infections caused by *C. albicans*, has emerged as a major complication [11,12,13,14]. This is concerning because candidemia in COVID-19 patients is associated with delayed diagnosis owing to overlapping clinical features, high rates of antifungal resistance in non-*albicans* species, and markedly increased morbidity and mortality rates [15,16]. Therefore, candidemia has become a significant secondary infectious complication observed primarily in hospitalized COVID-19 patients treated in ICUs, posing a marked challenge to infection control and patient outcomes during the pandemic.

The rising global burden of invasive fungal infections has increased the importance of *C. albicans* in public health, especially among ICU patients, owing to its prevalence, diagnostic and treatment difficulties, and high mortality rates [17,18,19,20]. However, a knowledge gap still remains regarding the epidemiology, antifungal resistance burden, and risk factors for *C. albicans* infection. In particular, limited research has been conducted on such infections in Türkiye, leaving a critical lack of data to inform clinical and epidemiological strategies.

This study provides a quantitative assessment of the burden of *C. albicans* candidemia in Adana Province, Türkiye, and informs healthcare professionals to devise antifungal regimens based on these findings. Our previous studies, which highlighted that outbreaks of fluconazole-resistant (FLC-R) *C. parapsilosis* isolates in Türkiye have been pivotal in informing physicians to restrict azole use in impacted healthcare centers [11,21,22,23,24,25]. Similarly, the findings of the present study are expected to serve as a valuable resource for both healthcare providers and policymakers to shape health strategies.

## 2. Materials and Methods

### 2.1. Study Design

Sepsis was suspected in 171 patients with at least one blood culture positive for *C. albicans*, between 1 March 2019 and 28 February 2023, at Adana City Training and Research Hospital, Türkiye, a 1550-bed hospital, including 310 ICU beds. No additional inclusion or exclusion criteria were applied. Patients were divided into four groups based on the COVID-19 pandemic period: (i) pre-COVID-19 (March 2019–February 2020), (ii) 1st year post-COVID-19 (March 2020–February 2021), (iii) 2nd year post-COVID-19 (March 2021–February 2022), and (iv) 3rd year post-COVID-19 (March 2022–February 2023) [8,10,14].

### 2.2. Data Collection and Definitions

Epidemiological characteristics and risk factors of patients were evaluated retrospectively using hospital records. Variables included such as age, sex, hospitalization unit, COVID-19 status, diabetes mellitus (DM), use of hyperalimentation fluids, hemodialysis, steroid use, prematurity, antifungal use before and after candidemia, prior surgical interventions, hematological/oncological diseases, CVC placement, mechanical ventilation, and length of ICU stay.

A candidemia episode was defined as the isolation of *Candida* species in blood culture. Recurrent positive cultures within 30 days were considered the same episode, while those detected with an interval longer than 30 days were considered separate episodes [26,27,28,29]. Thirty-day mortality was defined as all-cause death within 30 days of the candidemia diagnosis [14,30].

### 2.3. Clinical Samples

Blood culture samples were incubated in the BD BACTEC™ FX (Becton Dickinson, Franklin Lakes, NJ, USA) system. Samples showing budding yeast cells on Gram stain were inoculated onto 5% sheep blood agar (Oxoid, Basingstoke, UK), eosin methylene blue agar (Oxoid), Sabouraud glucose agar (Oxoid), and CHROMagar Candida (CAC; CHROMagar, Paris, France) media and incubated aerobically at 35 ± 2 °C for 24–48 h [31,32]. The resulting yeast colonies were identified to the species level using matrix-assisted laser desorption/ionization time-of-flight mass spectrometry (MALDI-TOF MS; Bruker Biotyper; Bruker Daltonics, Bremen, Germany) [33,34].

### 2.4. Antifungal Susceptibility Testing (AFST)

AFST was performed using the broth microdilution method according to the Clinical & Laboratory Standards Institute (CLSI) M27-Ed4 [35] recommendations, and results were interpreted according to CLSI M27M44S [36]. *Candida krusei* ATCC 6258 and *C. parapsilosis* ATCC 22019 reference strains were used for quality control.

### 2.5. Statistical Analysis

Categorical data are summarized as numbers and percentages, and numerical data as mean ± standard deviation. In comparisons of categorical data between groups, the Pearson’s chi-square test or Fisher’s exact test was used when there were problems with the expected values. The normality of numerical data was assessed using the Shapiro–Wilk test; if the data were normally distributed, the *t*-test for independent groups was applied, otherwise, the Mann–Whitney U test was used. Mortality risk factors were examined using logistic regression, and survival times were analyzed using the Kaplan–Meier method and the log-rank tests. Variables affecting survival were determined using Cox regression analysis. All analyses were performed using SPSS 20.0, and *p* < 0.05 was considered statistically significant.

## 3. Results

### 3.1. General Characteristics of the Study Population and Mortality Rates

Of the 171 patients with *C. albicans* candidemia included in the study, 107 (62.6%) were male, and 64 (37.4%) were female, with a median age of 61 years (range: 0–90 years). The mortality rate was 61.7% in men and 75.0% in women (*p* = 0.074). Mortality increased significantly with age: 72.4% among patients aged 40–64 years, 81% among those aged 65–79 years, and 91.7% among those aged ≥80 (*p* < 0.001). In the logistic and Cox regression analyses, age was found to increase the risk of mortality by 1.03 and 1.02 times, respectively (*p* < 0.001).

### 3.2. The Impact of Demographic and Clinical Characteristics on Mortality

In univariate analyses examining the relationship between the presence or absence of various clinical and demographic factors and mortality, mortality was significantly increased with ICU admission (75.8% vs. 35.9%, *p* < 0.001), COVID-19 positivity (80.4% vs. 57.9%, *p* = 0.008), DM (82.5% vs. 58.8%, *p* = 0.002), hemodialysis (87.0% vs. 59.2%, *p* = 0.001), steroid use (73.1% vs. 55.6%, *p* = 0.019), hyperalimentation (75.0% vs. 60.6%, *p* = 0.049), presence of a CVC (72.7% vs. 23.8%, *p* < 0.001), and mechanical ventilation (81.7% vs. 35.7%, *p* < 0.001). Although antifungal use before and after candidemia was not significant in univariate analysis, it was identified as an independent risk factor for mortality in the Cox regression analysis (HR = 3.47, 6.59, *p* < 0.001). Prematurity, previous surgical intervention, and hematological/oncological disease were not significantly related to mortality (*p* > 0.05). FLC was the most commonly used antifungal with mortality rates of 63.6% (*n* = 63) for FLC, 86.7% (*n* = 13) for echinocandin, 40% (*n* = 6) for FLC + echinocandin, 100% (*n* = 7) for amphotericin B (AMB), and 25% (*n* = 1) for FLC + AMB. Patients treated with FLC + AMB or FLC + echinocandin showed higher survival rates. Mortality rates by study periods were as follows: pre-COVID-19, 70.6%; 1st year post-COVID-19, 66%; 2nd year post-COVID-19, 70.3%; and 3rd year post-COVID-19, 61.7%. The highest mortality rates were observed during the pre-COVID-19 period, whereas the lowest mortality rate was during the 3rd post-COVID-19 year. However, no statistically significant difference was found in mortality rates across the study periods (*p* = 0.759).

The relationships between the demographic and clinical characteristics of the study population and mortality are presented in Table 1.

### 3.3. The Effect of Time-Dependent Clinical Parameters on Mortality

Differences in continuous variables between the survivor and non-survivor groups were evaluated using the *t*-test and Mann–Whitney U test (Table 2). The mean age of the non-survivor group was 58.8 years (SD = 21.8), whereas that of the survivor group was 35.6 years (SD = 29.8), and a significant difference was found between age and mortality (*p* < 0.001). The average hospital stay was 49.9 days (SD = 41.5) and 35.3 days (SD = 26.3) for non-survivor and survivor groups, respectively, and this difference was statistically significant (*p* = 0.021). No significant differences in mortality were observed based on the duration of candidemia (*p* = 0.815), length of stay in the ICU (*p* = 0.611), days with a CVC (*p* = 0.234), or duration of mechanical ventilation (*p* = 0.144) after ICU admission.

### 3.4. Results of Evaluating Independent Risk Factors Determining Mortality

The independent factors predicting mortality were examined using logistic and Cox regression analyses. According to logistic regression, age (OR = 1.03, 95% CI = 1.02–1.04, *p* < 0.001), ICU admission (OR = 2.05, 95% CI = 1.08–3.85, *p* = 0.027), steroid use (OR = 2.56, 95% CI = 1.55–4.23, *p* < 0.001), and hyperalimentation fluid use (OR = 2.48, 95% CI = 1.38–4.46, *p* = 0.002) were found to increase mortality risk (Table 3).

According to the Cox regression analysis, the factors most strongly associated with increased risk of mortality were: age (HR = 1.02, 95% CI = 1.00–1.03, *p* < 0.001), ICU admission (HR = 2.28, 95% CI = 1.26–4.12, *p* = 0.006), prior antifungal use before candidemia (HR = 3.47, 95% CI = 2.03–5.92, *p* < 0.001), and the absence of antifungal treatment after candidemia (HR = 6.59, 95% CI = 3.81–11.38, *p* < 0.001) (Table 4).

### 3.5. Survival Analysis in Patients with Candidemia (Kaplan–Meier Analysis)

Kaplan–Meier analysis was used to evaluate the survival of 171 patients after their first candidemia episode. The total mortality rate was 66.7% (*n* = 114), while the survival rate was 33.3% (*n* = 57). The 30-day mortality rate was 64% for all patients, 77.9% (*n* = 46) for COVID-19-positive patients, and 59.2% (*n* = 95) for COVID-19-negative patients. The mean survival time was 34.3 day (95% CI = 25.6–42.9), and the median survival time was 15.2 day (95% CI = 11.4–18.9). The 10-, 20-, 30-, and 40-day survival rates after candidemia onset were 61.4%, 44.4%, 34.0%, and 25.2%, respectively. The 30-day survival probabilities were 22.1% for COVID-19-positive patients and 40.8% for negative patients (Table S1).

Details of 171 patients with candidemia included in the study, their relationships with the clinics where they were treated, and survival probabilities are presented in Figure S1; the relationship between antifungal use after candidemia and survival probability is shown in Figure S2.

### 3.6. AFSTs

The in vitro susceptibilities of 171 *C. albicans* isolates to seven antifungals were examined using the CLSI broth microdilution method. The minimum inhibitory concentration (MIC) ranges for AMB, anidulafungin (AND), caspofungin (CAS), FLC, micafungin (MCF), posaconazole (POS), and voriconazole (VRC) were 0.5–1, 0.015–0.03, 0.03–0.25, 0.125–2, 0.015–0.03, 0.03–0.06, and 0.03–0.125 µg/mL, respectively. AND, MCF, POS, and VRC had MIC_90_ values of 0.03 µg/mL and were determined to have the highest antifungal activity. MIC_50_ and MIC_90_ values for AMB, AND, CAS, FLU, MCF, POS, and VRC were found to be 0.5–1, 0.015–0.03, 0.25, 0.125–0.25, 0.03, 0.03, and 0.03 µg/mL, respectively. No MIC value indicating resistance was detected in any of the isolates, and all were found susceptible strains. MIC distribution results for *C. albicans* isolates against the tested antifungal drugs are shown in Figure S3. MIC ranges for quality control strains are provided in Table S2.

## 4. Discussion

The present study quantitatively revealed the burden of *C. albicans* candidemia in the Adana region. The data we used as the basis for our study are crucial for the clinical management of this infection, especially for identifying effective risk factors at the local level and the AFST profile. Although all *C. albicans* isolates in Adana were susceptible to all antifungals (AMB, azoles, and echinocandins), the high mortality rate (66.7%) observed in cases of candidemia is concerning. While delays in diagnosis and treatment were evident, the patients’ immune status and preexisting risk factors may also have directly contributed to this delay. In our study, when factors affecting mortality based on survival time were evaluated, increasing age was found to be a strong prognostic marker for candidemia-related mortality. The significant increase in mortality, especially in individuals aged >40 years, indicates that this patient group requires closer monitoring. The significantly higher mortality rate (75.8%) among patients hospitalized in the ICU compared with those in the general ward (35.9%) supports the direct relationship between the need for intensive care, disease severity, and poor prognosis. Additionally, the significant increase in mortality risk associated with steroid treatment and hyperalimentation fluid use suggests that these treatments should be used more cautiously in high-risk patients.

The significant increase in mortality rates with age and the fact that mortality rates in patients aged ≥65 exceed 80% support the conclusions of international meta-analyses and cohort studies that have highlighted advanced age as an independent risk factor for mortality in candidemia [8,20,37,38,39,40,41,42]. Age is often associated with increased comorbidities, polypharmacy, reduced physiological reserves, and weakened immune responses in this context. Thus, age should also be included as a fundamental variable in risk classification for candidemia.

In our study, ICU hospitalization was an independent risk factor for mortality, which is consistent with the literature [6,18,43,44]. Strict infection control, active surveillance, and rapid empirical antifungal treatment are important for patients in the ICU. Steroid use and hyperalimentation were found to be independent risk factors for mortality, consistent with the literature [18,45,46,47,48,49]. Although both interventions are necessary in critical care, they increase the risk of candidemia by leading to immunosuppression; therefore, clinicians should consider this risk during treatment.

Antifungal use before candidemia was identified as another independent risk factor for increased mortality. This suggests that a history of antifungal treatment may indicate more severe clinical presentation, exposure to resistant strains, or serious underlying comorbidities [47]. In contrast, the absence of antifungal treatment after candidemia was also found to increase mortality 6.59-fold. The literature also shows that timely and appropriate antifungal therapies reduce mortality [47,50,51,52,53]. These findings reveal that early and effective antifungal treatment is a critical and modifiable factor for improving survival.

Mortality due to candidemia in COVID-19 positive patients was significantly higher (80.4%) compared to COVID-19 negative patients (57.9%) (*p* = 0.008), and the 30-day survival rates were significantly lower (22.1% and 40.8%, respectively). This may be associated with dysregulation of the immune response to COVID-19, lung damage, and increased invasive interventions. Similarly, the literature has highlighted high mortality rates and delays in antifungal treatment during COVID-19 infection [12,38]. Based on our findings, developing early diagnostic approaches, promptly initiating antifungal treatment, and optimizing infection control measures are critically important for reducing mortality, especially in high-risk COVID-19 patients.

In our study, DM (82.5%, *p* = 0.002), hemodialysis (87%, *p* = 0.001), CVC implantation (72.7%, *p* = 0.001), and mechanical ventilation (81.7%, *p* = 0.001) were significantly associated with mortality. These findings are consistent with the literature [6,18,48,54,55,56,57,58,59,60,61]. Given that patients with DM, undergoing hemodialysis, or receiving CVC or mechanical ventilation are more vulnerable to candidemia, effective management of these comorbidities and candidemia prophylaxis strategies should be evaluated.

The median survival time was 15.17 day, and the decrease in survival probability from 61.4% to 25.2% in the first 40 day indicated that a significant proportion of candidemia-related deaths occurred during the early stages of the disease. These findings reinforce the clinical significance of (i) early diagnosis, (ii) prompt initiation of treatment, and (iii) rapid identification of high-risk patients. We found a significant difference between length of hospital stay and mortality (*p* = 0.021). The longer hospital stays of surviving patients compared with those of non-survivors (49.9 vs. 35.3 day, *p* = 0.018) also reflected a common paradox in serious infections. Length of stay should not be considered an independent risk factor but rather an indirect indicator of survival among patients who have passed the critical period.

Our study showed that all 171 *C. albicans* isolates from patients with candidemia retained their susceptible properties against all antifungals. Our findings are consistent with those of a multicenter study conducted across Türkiye [62]. Many other studies have shown that *C. albicans* has a high sensitivity profile to antifungals, with resistance to FLC generally remaining below 5% [19,63,64,65].

However, the situation is different for non-*albicans Candida* species, which have developed antifungal resistance, making them a serious global threat. Increased resistance rates have been observed in *C. parapsilosis*, *C. tropicalis*, *C. glabrata*, and *C. auris*. In multicenter studies conducted in Türkiye, it has been reported that FLC-R in *C. parapsilosis* isolates reached 26–28%, while significant rates of azole resistance were also found in *C. tropicalis* (6–9%) and *C. glabrata* (4%) isolates [22,23,24,66,67,68]. In our recent multicenter study, *C. parapsilosis* isolates from our country showed resistance rates of 26.7% and 2.1% against FLC and echinocandin, respectively. The fact that four isolates showed multidrug resistance indicates that treatment options may become increasingly limited [21]. Our findings indicate regional epidemiological differences compared to FLC-R *C. parapsilosis* outbreaks reported in the literature.

These findings indicate that antifungal resistance in *C. albicans* does not yet pose a clinically significant threat; however, the increasing rates of resistance in non-*albicans Candida* species, both in Türkiye and worldwide, are seriously concerning. Therefore, it is critical to perform AFSTs as part of routine testing in cases of candidemia, strengthen molecular epidemiological surveillance, and develop strategies to prevent the development of resistance.

While resistance in *C. albicans* was not observed and FLC was the most commonly used antifungal agent, the mortality rate was high (63.6%). The mortality rate was also high in patients treated with echinocandins (86.7%) or AMB (100%). Factors associated with high mortality (66.7%), such as delays in diagnosis or treatment initiation, the patient’s underlying immune status, and the presence of serious risk factors, suggest that these factors directly contribute to poor prognosis. Even if the medications are effective, poor outcomes persist owing to systemic problems in healthcare delivery and the extreme vulnerability of the patient population. The better survival in patients receiving combination therapy (FLC + AMB, FLC + echinocandin) suggests that treatment success may be enhanced with combination agents rather than a single agent. To improve outcomes, a multifaceted approach focusing on early diagnosis and rapid supportive care, in addition to the use of effective antifungals, is needed.

Our results show that patients undergoing steroid treatment, receiving hyperalimentation therapy, using antifungal treatment before candidemia, and not receiving antifungal treatment after infection should be considered at high risk for candidemia. Diagnostic and treatment processes should be accelerated in this population when candidemia is suspected. When candidemia is diagnosed, initiating appropriate antifungal treatment immediately is vital for improving patient survival. The major limitations of our study are the retrospective nature of the research, the absence of molecular typing for *C. albicans*, the focus on *C. albicans* cases alone, and the limited generalizability of the findings due to the single-center design.

In the future, a larger series of phenomena and multi-center studies should be conducted. Studies incorporating standardized severity of illness scores, such as APACHE II and SOFA, would allow for better control of confounding factors, such as indication bias, and a more accurate assessment of the true impact of antifungal use. Additionally, prospective studies should be conducted to evaluate the impact of the timing of antifungal treatment initiation and optimal dosage on survival after candidemia. Qualitative studies or surveys could provide an understanding of the decision-making processes of clinicians when selecting antibiotics and antifungals, as well as the factors influencing these decisions. Using epidemiological data-based surveillance practices to determine and monitor the antifungal resistance profiles of *C. albicans* isolates in the Adana region and updating empirical treatment guidelines will be necessary to increase awareness of fungal infections and continuously monitor the dynamics that may contribute to infection.

## Figures and Tables

**Table 1:** Relationship between demographic and clinical characteristics of the study population and mortality.

Feature	Category	Survivor (*n* = 57)	Non-Survivor (*n* = 114)	*p*-Value
Gender	Male	41 (38.3%)	66 (61.7%)	0.074
	Female	16 (25%)	48 (75%)	
Age group	0–2	16 (69.6%)	7 (30.4%)	<0.001
	3–17	7 (70%)	3 (30%)	
	18–39	6 (60%)	4 (40%)	
	40–64	16 (27.6%)	42 (72.4%)	
	65–79	11 (19%)	47 (81%)	
	80+	1 (8.3%)	11 (91.7%)	
Clinics	Intensive care unit	32 (24.2%)	100 (75.8%)	<0.001
	Service	25 (64.1%)	14 (35.9%)	
Presence of COVID	Negative	40 (42.1%)	55 (57.9%)	0.008
	Positive	9 (19.6%)	37 (80.4%)	
Diabetes mellitus	No	47 (41.2%)	67 (58.8%)	0.002
	Yes	10 (17.5%)	47 (82.5%)	
Hyperalimentation fluid uses	No	39 (39.4%)	60 (60.6%)	0.049
	Yes	18 (25%)	54 (75%)	
Hemodialysis	No	51 (40.8%)	74 (59.2%)	<0.001
	Yes	6 (13%)	40 (87%)	
Steroid use	No	28 (44.4%)	35 (55.6%)	0.019
	Yes	29 (26.9%)	79 (73.1%)	
Premature	No	53 (32.3%)	111 (67.7%)	0.172
	Yes	4 (57.1%)	3 (42.9%)	
Antifungal use before candidemia	No	53 (35.8%)	95 (64.2%)	0.081
	Yes	4 (17.4%)	19 (82.6%)	
Previous surgical interventions	No	20 (26.3%)	56 (73.7%)	0.082
	Yes	37 (38.9%)	58 (61.1%)	
Hematological/oncological diseases	No	46 (37.1%)	78 (62.9%)	0.090
	Yes	11 (23.4%)	36 (76.6%)	
Central venous catheterization	No	16 (76.2%)	5 (23.8%)	<0.001
	Yes	41 (27.3%)	109 (72.7%)	
Mechanical ventilation	No	36 (64.3%)	20 (35.7%)	<0.001
	Yes	21 (18.3%)	94 (81.7%)	
Antifungal treatment (after candidemia)	No	6 (21.4%)	22 (78.6%)	0.144
	FLC	36 (36.4%)	63 (63.6%)	
	Echinocandin	2 (13.3%)	13 (86.7%)	
	FLC + echinocandin	9 (60%)	6 (40%)	
	AMB	0 (0%)	7 (100%)	
	FLC + AMB	3 (75%)	1 (25%)	
	CAS + AMB	0 (0%)	1 (100%)	
	FLC + echinocandin + AMB	1 (50%)	1 (50%)	
Study period	Pre-COVID	5 (29.4%)	12 (70.6%)	0.759
	1st year post-COVID	10 (33.3%)	20 (66.7%)	
	2nd year post-COVID	19 (29.7%)	45 (70.3%)	
	3rd year post-COVID	23 (38.3%)	37 (61.7%)	

**Table 2:** Relationship between time-dependent clinical parameters and mortality (results of *t*-test and Mann–Whitney U Test).

Feature	Clinical Outcome	*n*	Average	Standard Deviation	Median	*p*-Value
Age	Survivors	57	35.6	29.835	60.69	<0.001
	Non-survivors	114	58.8	21.845	98.65	
ICU length of stay (days)	Survivors	42	40.6	46.753	78.92	0.611
	Non-survivors	109	29.5	26.456	74.88	
Hospital length of stay (days)	Survivors	57	49.9	41.493	98.39	0.021
	Non-survivors	114	35.3	26.339	79.80	
Central venous catheter (days)	Survivors	41	39.7	40.498	82.38	0.234
	Non-survivors	109	29.0	24.902	72.91	
Mechanical ventilation (days)	Survivors	21	31.2	36.761	67.60	0.144
	Non-survivors	94	17.7	19.553	55.86	

**Table 3:** Independent risk factors associated with mortality (Logistic regression analysis).

Variables	*p*-Value	Odds Ratio (OR)	95% Confidence Interval for OR
Age	**<0.001**	1.03	1.02–1.04
ICU admission	**0.027**	2.05	1.08–3.85
Steroid use	**<0.001**	2.56	1.55–4.23
Use of hyperalimentation fluids	**0.002**	2.48	1.38–4.46

**Table 4:** Independent predictors of mortality based on survival time (Cox regression analysis).

Variables	*p*-Value	Hazard Ratio (HR)	95% Confidence Interval for HR
Age	**<0.001**	1.02	1.01–1.03
ICU admission	**0.006**	2.28	1.26–4.12
Antifungal use before candidemia	**<0.001**	3.47	2.03–5.92
No antifungal use after candidemia	**<0.001**	6.59	3.81–11.38

## Data Availability

The original contributions presented in this study are included in the article/Supplementary Material. Further inquiries can be directed to the corresponding author.

## Supplementary Materials

The following supporting information can be downloaded at: https://www.mdpi.com/article/10.3390/jof11110788/s1, Figure S1: Relationship between the clinics where candidemia cases were treated and the probability of survival; Figure S2: Relationship between antifungal use and survival probability after candidemia; Figure S3: MIC values (µg/mL) for *Candida albicans* isolates against the tested antifungal drugs. Abbreviations: AMB, amphotericin B; AND, anidulafungin; CAS, caspofungin; FLC, fluconazole; MCF, micafungin; POS, posaconazole; VRC, voriconazole; Table S1: Kaplan–Meier survival analysis results; Table S2: CLSI MIC QC (µg/mL) ranges of organisms used as quality control strains in this study.

## Abbreviations

The following abbreviations are used in this manuscript:

AFST	antifungal susceptibility testing
AMB	amphotericin B
AND	anidulafungin
CAS	caspofungin
CLSI	Clinical & Laboratory Standards Institute
CVC	central venous catheter
DM	diabetes mellitus
FLC	fluconazole
HR	hazard ratio
ICU	intensive care unit
MCF	micafungin
MIC	minimum inhibitory concentration
N/A	not available
POS	posaconazole
VRC	voriconazole
