# Supplementary material for: Epidemiological Characteristics and Mortality Predictors of Candidemia Due to Candida albicans: A Single-Center Experience from Türkiye
Source: J Fungi (Basel). 2025 Nov 2;11(11):788. doi: 10.3390/jof11110788 (PMC12653465; doi:10.3390/jof11110788)
Supplement: Supplementary file 1 [file jof-11-00788-s001.zip › jof-3919411-supplementary.pdf]

**Table S1.** Kaplan-Meier survival analysis results.

| Measurement | Estimate | Standard | 95% Confidence | 95% Confidence Interval |
|---|---|---|---|---|
| Average survival (d) | 34.3 | 4.4 | 25.6 | 42.9 |
| Median survival (d) | 15.2 | 2.0 | 11.4 | 18.9 |

**Table S2.** MIC QC (µg/mL) ranges of organisms used as quality control strains in this study.

| Antifungal Agent | *C. krusei* ATCC 6258 | *C. parapsilosis* ATCC 22019 |
|---|---|---|
| Amphotericin B | 0.5–2 | 0.25–1 |
| Anidulafungin | 0.03–0.12 | 0.25–1 |
| Caspofungin | 0.5–1 | 0.5–1 |
| Fluconazole | 16 | 0.5–2 |
| Micafungin | 0.125–0.25 | 0.5–1 |
| Posaconazole | 0.06 | 0.03 |
| Voriconazole | 0.06 | 0.016 |

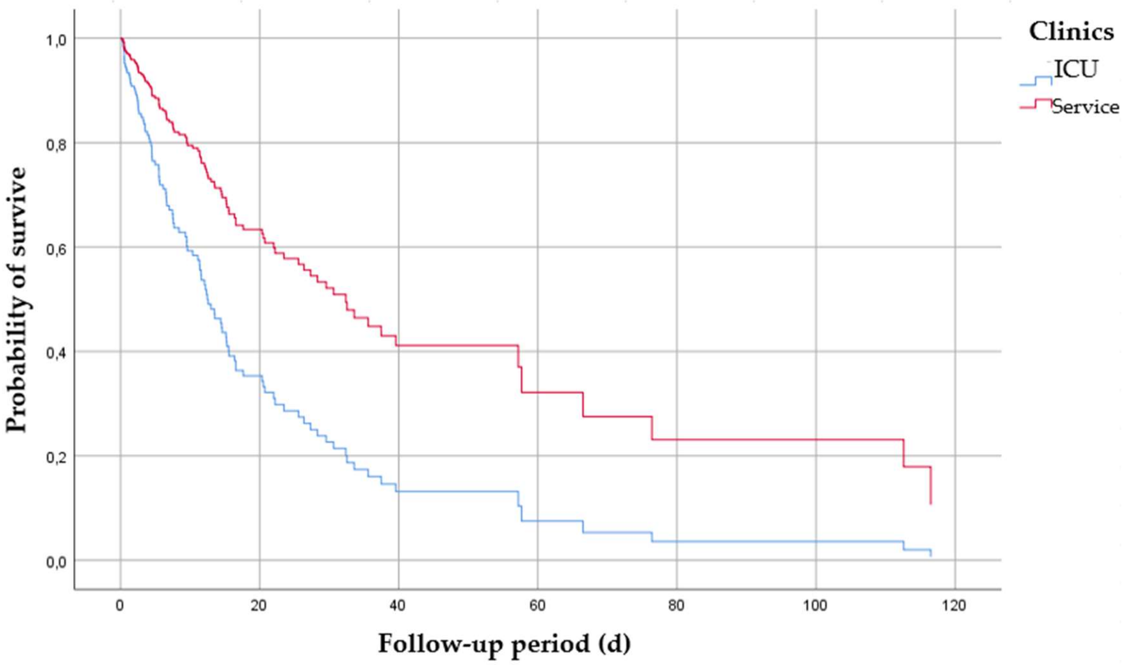


**Figure S1** Relationship between the clinics where candidemia cases were treated and the probability of survival.

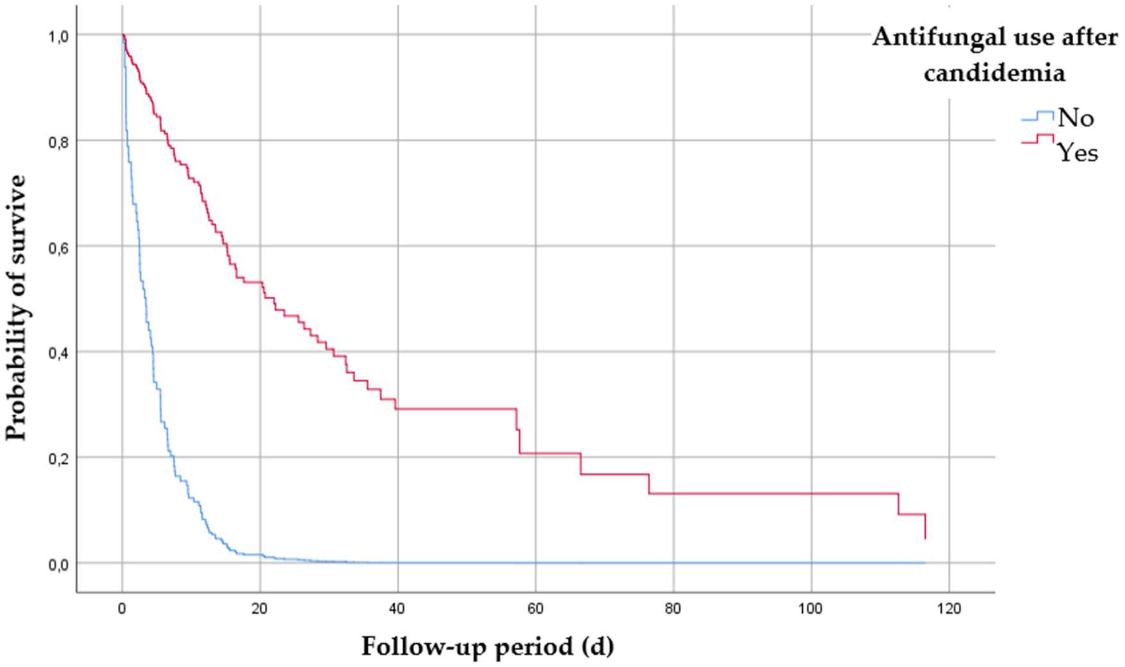


**Figure S2** Relationship between antifungal use and survival probability after candidemia.

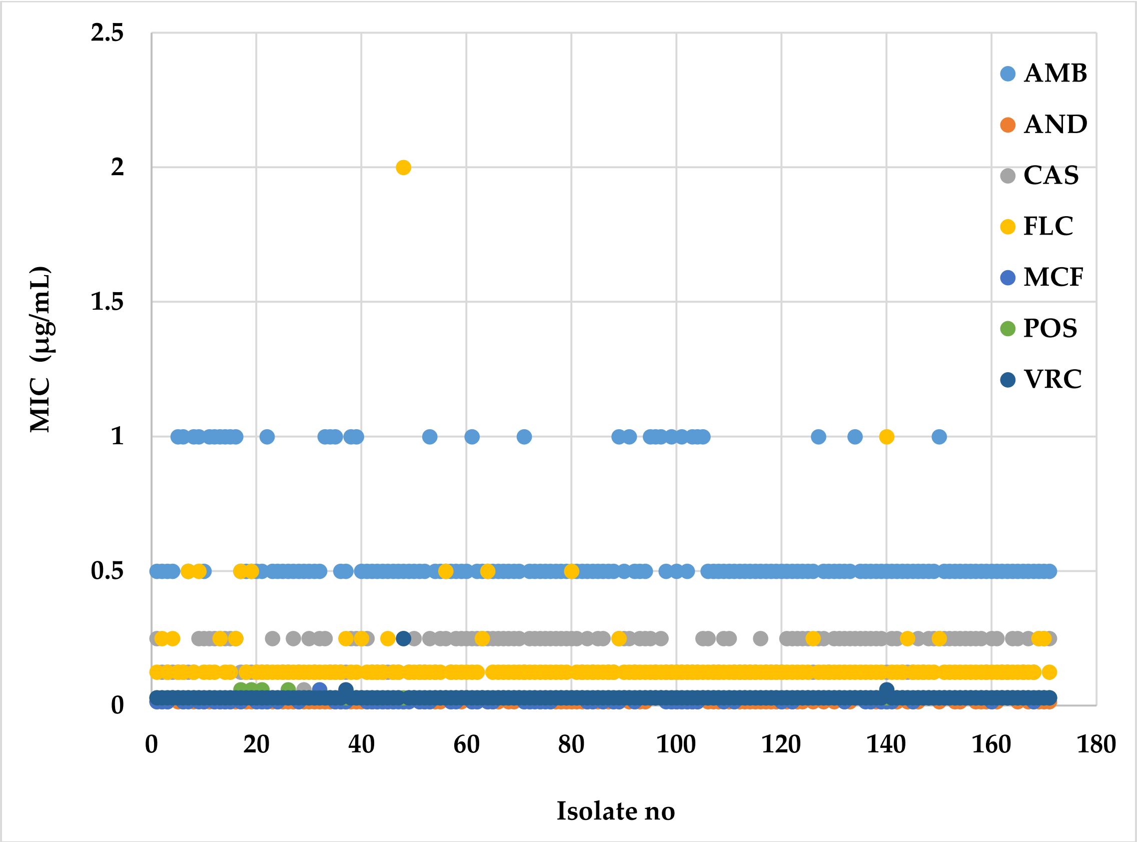


**Figure S3** MIC values (µg/mL) for *Candida albicans* isolates against the tested antifungal drugs. Abbreviations: AMB, amphotericin B; AND, anidulafungin; CAS, caspofungin; FLC, fluconazole; MCF, micafungin; POS, posaconazole; VRC, voriconazole.
